# Association of Serum Ferritin With Growth and Endocrine Function in Thalassemia Major Children in North India: An Observational Study

**DOI:** 10.7759/cureus.74885

**Published:** 2024-11-30

**Authors:** Ganesh Verma, Ramesh Chand, Meraz Anjum, Dushyant Rastogi, Nishant Sharma, Swalpa Verma, Imran Ahmed Khan

**Affiliations:** 1 Pediatrics, Uttar Pradesh University of Medical Sciences, Saifai, IND; 2 Dentistry, Community Health Centre, Khairagarh, IND; 3 Community Medicine, Baba Raghav Das Medical College, Gorakhpur, IND

**Keywords:** anemia, beta-thalassemia, blood transfusion, chelation therapy, iron overload

## Abstract

Background

Thalassemia is the most common form of hereditary anemia caused by the impaired synthesis of one of the two globin chains in hemoglobin. A decrease in beta-globin chains occurs in beta-thalassemia, resulting in a relative excess of alpha-globin chains. Thalassemia major is the severe form of thalassemia, which requires frequent blood transfusions for survival. Consequently, the natural course of the disease is affected by blood transfusion-related side effects. Repeated blood transfusions lead to the accumulation of iron in tissues such as the liver, heart, and endocrine glands. Serum ferritin is a biomarker of iron overload. Endocrinopathies are among the most frequently observed complications in thalassemia. Early recognition and treatment are important in order to prevent late irreversible sequelae and improve the quality of life of these patients. This study was conducted to evaluate growth parameters and endocrine function in children with thalassemia major and their relation with serum ferritin.

Methods

This prospective observational study included all patients between the age groups six months and 14 years with transfusion-dependent thalassemia. We included 62 children admitted during the study period fulfilling eligibility criteria. The data was analyzed using descriptive statistics and making comparisons among various groups. Spearman correlation analysis was done to assess the correlation between serum ferritin and thyroid hormone. The difference of means across the groups was tested with the Mann-Whitney U test for two groups and the Kruskal-Wallis test for more than two groups.

Results

The mean age of the study participants was 5.66 ± 3.77 years, with the largest group consisting of children aged one to three years, comprising 40.3% of the participants. The majority of participants were boys. This study showed a high prevalence of endocrinopathies in transfusion-dependent thalassemic patients. The most common endocrinopathy was short stature (37.1%), followed by impaired glucose tolerance (28.6%), subclinical hypothyroidism (14.5%), and parathyroid dysfunction (14.5%). Overt diabetes and pubertal delay were not seen. A statistically significant association of ferritin was found with age (p < 0.001), stature (p = 0.001), thryroid-stimulating hormone (TSH) (p = 0.004), and parathyroid function (p = 0.006).

Conclusions

The prevalence of endocrinopathies in present transfusion-dependent thalassemic cohorts was considerably high, presenting as short stature, impaired glucose tolerance, hypoparathyroidism, and subclinical hypothyroidism. The study showed a weak positive correlation of endocrinopathies with serum ferritin levels. Hence, irrespective of serum ferritin levels, patients with transfusion-dependent thalassemia can have a considerably high prevalence of endocrine complications.

## Introduction

Thalassemia, the most common form of hereditary anemia, is caused by the impaired synthesis of one of the two globin chains in hemoglobin [1]. This disorder has been found to be highly prevalent in tropical and sub-tropical regions of the world (e.g., Southeast Asia, the Mediterranean area, the Indian subcontinent, and Africa), where the estimated prevalence rates are 12%-50% in the case of alpha thalassemia and 1%-20% for beta thalassemia [2]. The overall prevalence of the thalassemia trait in India is 2.78%, with higher burdens in northern and western states [3,4]. Beta thalassemia syndromes result from a decrease in beta-globin chains, which results in a relative excess of alpha-globin chains.

The life expectancy of thalassemic patients has dramatically extended due to the availability of multiple transfusions and chelation therapy. The majority of children with severe forms of thalassemia, such as thalassemia major, are transfusion-dependent, requiring blood transfusions every 15-30 days [5]. The natural course of the disease is affected by transfusion side effects, which need to be monitored and treated [6]. Iron overload resulting in end-organ damage and blood-borne infectious agents still represent the principal causes of morbidity and mortality. Due to repeated blood transfusions, iron accumulates in tissues such as the liver, heart, and endocrine glands, as these organs have high levels of transferrin receptors. Serum ferritin is a valuable parameter in managing thalassemia, particularly for monitoring iron overload and guiding chelation therapy. A serum ferritin level >1000 ng/ml indicates iron overload, which is associated with harmful consequences such as organ damage and increased mortality [7].

Endocrinopathies are among the most frequently observed complications in thalassemia. Hormone secretion disorders among endocrine organs affected by iron deposition include the pituitary, adrenal, pancreas, thyroid, and parathyroid glands leading to glucose intolerance, and gonadal, thyroid, and parathyroid dysfunctions [8]. Growth retardation in thalassaemic children is not only seen in pubertal periods but also in the infantile and pre-pubertal periods [9]. Gonadal dysfunction is a major complication occurring in thalassemia due to gonadal iron deposition, as confirmed by multiple gonadal and pituitary-gonadal function studies [10]. Factors contributing to the development of impaired glucose tolerance or overt diabetes include chronic iron overload in the pancreas, causing impaired insulin excretory function [11]. Both primary effects as a result of iron deposition in the thyroid gland as well as secondary effects due to pituitary dysfunction have been observed in the affected children.

Overt clinical hypothyroidism develops in a minority of patients, that is, around 5%, whereas a larger percentage develops subclinical primary hypothyroidism as depicted by normal tetraiodothyronine (T4) and triiodothyronine (T3) levels but high thyroid-stimulating hormone (TSH) levels [12]. The development of hypoparathyroidism is mainly attributed to poor compliance with chelation therapy and elevated serum ferritin levels.

Hematopoietic stem cell transplantation (HSCT) is the only curative treatment for transfusion-dependent thalassemia, but it is not widely available in our country [13]. Thus, medical management of transfusion-dependent thalassemia, which includes multiple transfusion therapies, and management of endocrine dysfunctions appearing as a result of iron overload is a pressing medical challenge. Since endocrine complications have become a major problem in adolescence and adulthood, therefore early recognition and treatment are important in order to prevent late irreversible sequelae and improve the quality of life of patients.

With this background, the present study was conducted with the primary objective of evaluating growth parameters and endocrine function in children diagnosed with thalassemia major. The secondary objective was to investigate the impact of elevated serum ferritin on growth metrics and endocrine functions, focusing on markers of pubertal development, thyroid, and parathyroid functions. By identifying relationships between serum ferritin levels and growth or endocrine abnormalities, the study aimed to provide early interventions and improved management strategies for transfusion-dependent thalassemia major patients, ultimately enhancing their overall quality of life and clinical outcomes.

## Materials and methods

This prospective observational study was conducted in the Department of Pediatrics, Uttar Pradesh University of Medical Sciences (UPUMS) Saifai, Etawah. This study included all patients between the age groups of six months and 14 years with transfusion-dependent thalassemia admitted to the Department of Pediatrics at UPUMS, Saifai, Etawah.

Sample size 

We included 62 children admitted during the study period fulfilling eligibility criteria. Thalassemia major is a relatively rare condition, especially among pediatric populations. A sample size of 62 is practical and feasible within this specific population and region. Studies in similar domains have reported moderate effect sizes between ferritin levels and clinical outcomes, indicating that a sample size of 62 should be sufficiently sensitive to detect these relations. Ensuring comprehensive data collection on growth and endocrine markers may limit the scope for larger sample sizes, as each participant requires detailed testing for a range of outcomes (e.g., hormone levels and growth measurements). Previous studies on similar topics (e.g., the relationship between iron overload and endocrine or growth abnormalities in thalassemia patients) have often used sample sizes in the range of 50-100 participants [6,9]. This study's sample size ensures optimal use of available resources while achieving meaningful insights.

Inclusion and exclusion criteria

Children aged six months to 14 years with confirmed diagnoses of thalassemia major, undergoing regular blood transfusions, and willing to give informed and written consent for participation in the study were included. Children with other genetic or chronic illnesses and children on hormonal therapy that could affect growth and endocrine function were excluded.

Data collection

Data collection was conducted from the Thalassemia Care Center, UPUMS, Saifai, Etawah in North India, from December 2022 to May 2024. An informed written consent was obtained from parents/legal guardians of the patients eligible for participation in the study after approval from the institutional ethics committee of UPUMS, Saifai, Etawah. They were informed about the procedure in detail before the commencement of the study. Confidentiality and privacy were ensured. Demographic information like age and gender was collected. Growth assessment - measurements of height, stature, and bone age - was done. Evaluation of thyroid and parathyroid function was done with serum hormone levels assessed via blood tests. Serum ferritin levels of participants were obtained as a surrogate marker for iron overload.

Statistical analysis

The data collected were loaded in Microsoft Excel (Microsoft Corp, Redmond, WA) and coded. The analysis was done in IBM SPSS Statistics version 23 (IBM Corp, Armonk, NY). The data was analyzed using descriptive statistics and making comparisons among various groups. Categorical data was summarized as proportions and percentages, and quantitative data was summarized as mean ± standard deviation (SD). Data normality was found not normal by the Shapiro-Wilk test. Spearman correlation analysis was to study the correlation between serum ferritin and thyroid hormone. The difference of means across the groups was tested with the Mann-Whitney U test for two groups and the Kruskal-Wallis test for more than two groups. A two-sided p<0.05 was considered statistically significant.

## Results

**Figure 1:**
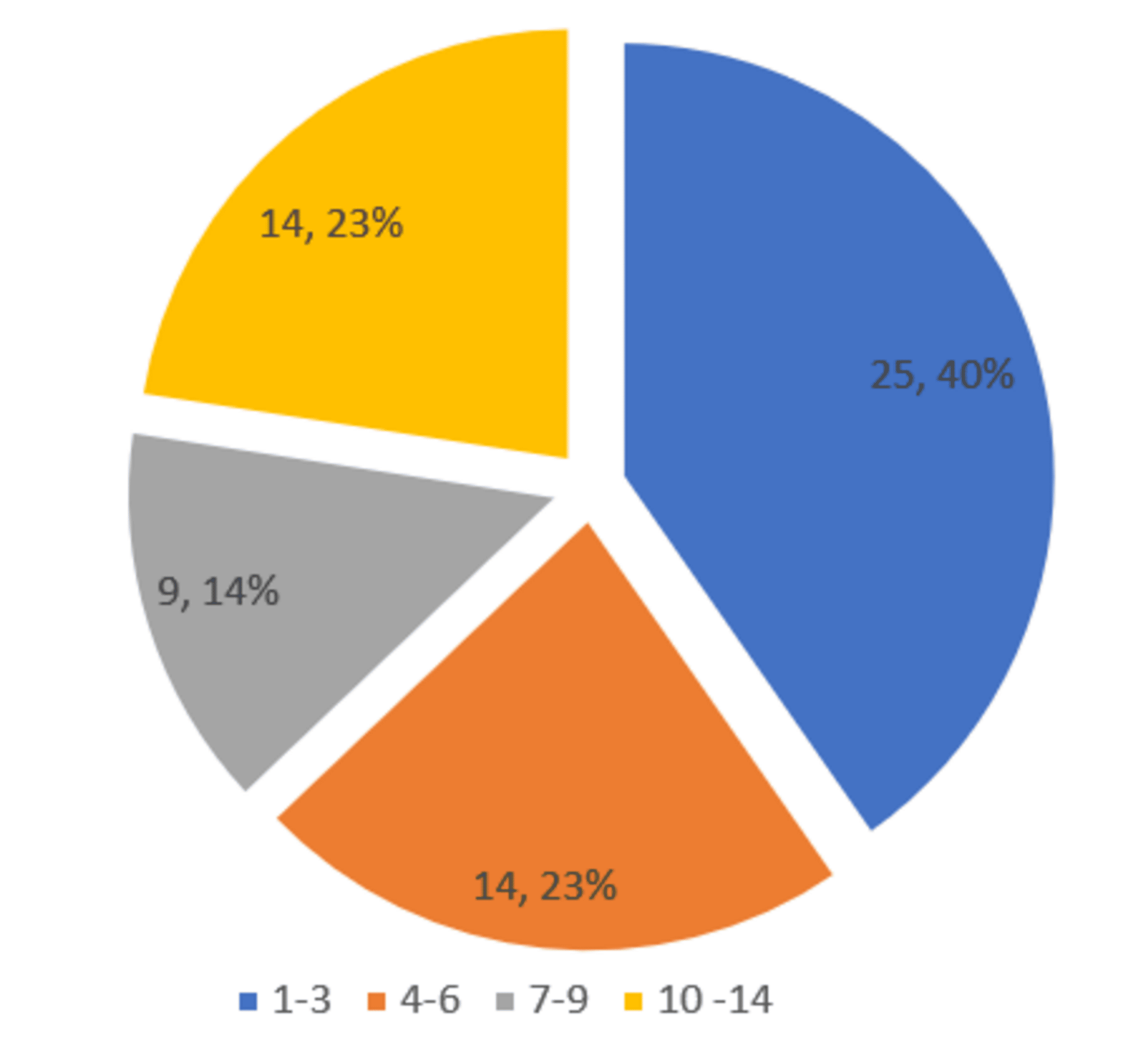
Age groups of participants (n = 62)

**Figure 2:**
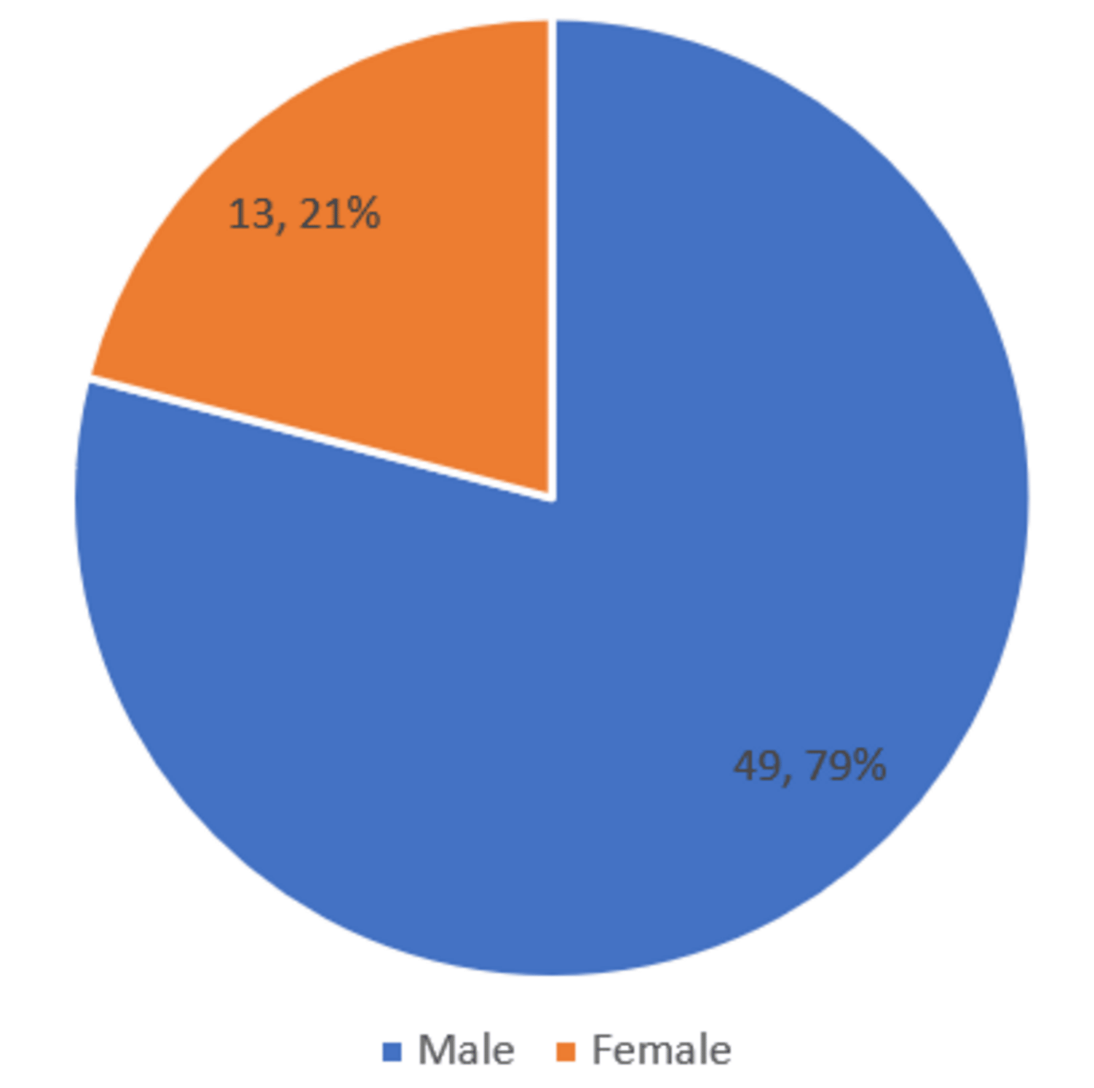
Gender distribution of participants (n = 62)

This observational study included 62 transfusion-dependent thalassemic children aged between six months and 14 years. Out of the 62 patients, the largest age group consisted of patients aged one to three years, comprising 25 (40.3%), and 49 (79%) were boys, as shown in Figures 1, 2.

Table 1 shows the clinical and biochemical characteristics of the participants. The most common endocrinopathy in transfusion-dependent thalassemia patients was short stature (37.1%), followed by impaired glucose tolerance (28.6%), subclinical hypothyroidism (14.5%), and parathyroid dysfunction (14.5%). Overt diabetes and pubertal delay were not detected in any of the patients.

**Table 1:** Clinical and endocrine characteristics of the participants (n = 62) TSH, thyroid stimulating hormone

Clinical and endocrine characteristics		Number (%)
Stature	No short stature	39 (62.9)
	Short stature	23 (37.1)
Bone age	Not delayed	52 (83.9)
	Delayed	10 (16.1)
Glucose tolerance	Not impaired	10 (71.4)
	Impaired	4 (28.6)
TSH	Elevated	49 (79)
	Normal	13 (21)
Thyroid dysfunction	No	53 (85.5)
	Yes	9 (14.5)
Parathyroid dysfunction	No	53 (85.5)
	Yes	9 (14.5)

The serum ferritin showed a strong positive correlation with age (r=0.688, p<0.001) and a weak positive correlation with TSH level (r=0.303, p=0.017), which were statistically significant (Table 2). In contrast, T3 and T4 demonstrated a weak, non-significant correlation with ferritin.

**Table 2:** Correlation between serum ferritin and participant’s characteristics TSH, thyroid stimulating hormone; T3, triiodothyronine; T4, tetraiodothyronine Spearman’s correlation was used to obtain the p-value.

Participant’s characteristics	Ferritin	
	Correlation coefficient	p-value
Age	.688	< .001
TSH	.303	.017
T3	.056	.663
T4	-.177	.170

Table 3 presents the mean serum ferritin levels (ng/ml) across participant characteristics, along with their associated p-values. There was a statistically significant association of ferritin with age (p<0.001), stature (p=0.001), TSH (p=0.004), and parathyroid function (p=0.006). An increased level of serum ferritin was observed with increasing age. The relationship between serum ferritin and bone age, thyroid, and parathyroid functions was not statistically significant.

**Table 3:** Relationship of serum ferritin and participant’s characteristics across groups TSH, thyroid stimulating hormone (ng/ml) ^a^Kruskal-Wallis test; ^b^Mann-Whitney U test A serum ferritin level >1000 ng/ml indicates iron overload, which is associated with harmful consequences, such as organ damage and increased mortality.

Participant’s characteristics		Serum ferritin (ng/ml) (mean ± SD)	p-value
Age (years)	1-3	1277.5 ± 1586.7	<.001^a^
	4-6	2848.1 ± 1356.2	
	7-9	3796.2 ± 2551.0	
	10-14	4098.8 ± 2145.3	
Gender	Male	2650.9 ± 2354.6	.359^b^
	Female	2574.2 ± 1254.1	
Stature	No short stature	1952.8 ± 1653.8	.001^b^
	Short stature	3791.4 ± 2451.5	
Bone age	Not delayed	2492.5 ± 2147.7	.210^b^
	Delayed	3374.9 ± 2192.9	
TSH	Not elevated	2268.8 ± 2010.3	.004^b^
	Elevated	4014.5± 2231.6	
Thyroid function	Not impaired	2517.5 ± 2109.6	.358^b^
	Impaired	3325.8 ± 2465.3	
Parathyroid function	Not impaired	2436.0 ± 2223.8	.006^b^
	Impaired	3805.6 ± 1297.7	

## Discussion

Beta-thalassemia major is a severe hemolytic anemia requiring regular blood transfusions [14]. In the present study of transfusion-dependent beta-thalassemia major children between the ages of six months and 14 years, the mean age was 5.66 ± 3.77 years. The largest group consisted of children aged one to three years, making up 40.3% of the participants. The majority of participants were boys, which is similar to another study [9].

A considerable proportion of short stature (37.1%) was found in our study. Chronic anemia, transfusion-related iron overload, and noncompliance with chelation therapy may be the responsible factors. Chronic anemia may lead to hypoxia and poor growth, whereas iron overload in endocrine glands impairs hormone synthesis and release, manifesting as hypothyroidism, growth hormone deficiency, micronutrient deficiency, undernutrition, and psychological stress. Short stature was detected to be the most common endocrine abnormality occurring in 40.2% of participants in a study by Tan et al. in Malaysian children with transfusion-dependent thalassemia [15]. In another study of multi-transfused Indian thalassemia patients, it was found that 57.14% of patients were short [16]. The prevalence of short stature in our study was lower than observed in some previous studies. The reason could be different genetic make-up and different classification criteria used for defining short stature.

The prevalence of thyroid dysfunction was found to be 14.5%. Out of nine patients found to have thyroid dysfunctions, all the patients had subclinical hypothyroidism, that is, elevated TSH with normal T3 and T4 values. There were no cases of secondary hypothyroidism. The results of this study are comparable to the previous studies. Tan et al. demonstrated subclinical hypothyroidism in 13.4% of patients and overt hypothyroidism in 4.9% of patients [15]. In another study by Bordbar et al., it was found that 10.7 patients had subclinical hypothyroidism [17]. Thyroid dysfunction frequently develops in thalassemia, with subclinical primary hypothyroidism occurring in a greater majority, mainly attributed to free radical release and oxidative stress due to iron overload. Overt clinical hypothyroidism occurs in a minority [18]. Endocrine dysfunctions in thalassemia major are multifactorial. Chronic anemia, hypoxia, oxidative stress, repeated transfusions, and inflammation independently impact endocrine function. These factors can disrupt hormone production and the conversion of T4 to T3. Therefore, weak correlations suggest that while iron toxicity is significant, other pathophysiological processes might play significant roles in endocrine dysfunctions.

Out of 14 patients who were more than 10 years old, five (28.6%) were tested impaired on the oral glucose tolerance test (OGTT), while there were no cases of overt diabetes. In the study by Tan et al. [15], pre-diabetes mellitus and overt diabetes were present in 8.6% and 5.2% of the patients, respectively. Pancreatic dysfunction because of chronic overload takes time to develop and is thus a late complication usually observed in the second decade of life. In our study, the number of patients more than 10 years of age was less. So, more patients in this age group and further follow-up are required to comment on the exact prevalence of dysglycemia in thalassemic patients.

In transfusion-dependent thalassemic patients, parathyroid dysfunction is evident after the first decade of life. In this study, parathyroid dysfunction was evaluated in all 62 patients with a 14.5% prevalence. In a similar study, hypoparathyroidism was observed in 12.3% of the patients [14]. In another study, 13.2% of the patients were found to have hypoparathyroidism [17].

Pubertal development was assessed in children more than 10 years of age in this study. The absence of breast development in girls by the age of 13 years and testicular development by the age of 14 years in boys defined pubertal delay. In our study, there was no patient above the age group for defining pubertal delay. Impaired puberty was present in 71% of patients in the study by Najafipour et al. [19]. In a study by Merchant et al., it was found that 60% of patients had not attained puberty [16]. Sexual underdevelopment is a cause of serious concern in beta-thalassemia patients as it represents the delayed onset of puberty [20].

Our study exhibits that the prevalence of short stature, parathyroid dysfunction, and thyroid dysfunction increases with rising serum ferritin levels. A study on Turkish children did not demonstrate ferritin levels to be significantly correlated with endocrine complications [21].

Thus, it can be said that patients with transfusion-dependent thalassemia can have endocrine complications irrespective of normal serum ferritin levels and vice versa. Therefore, close monitoring for endocrine dysfunction is essential irrespective of serum ferritin levels to prevent long-term adverse outcomes and to improve the quality of life [22]. These findings could underscore the need for early interventions to manage iron overload in thalassemia major patients and prevent or mitigate growth and endocrine complications. Improving chelation therapy compliance is another major target for managing complications of iron overload [23].

The study has a few noticeable strengths. It provides valuable insights into the relationship between serum ferritin levels and both growth and endocrine function among a well-defined cohort of transfusion-dependent thalassemia major children. The study provides region-specific data for North India where thalassemia prevalence is significant that can be helpful in tailoring clinical practices. The use of robust statistical analyses to establish correlations adds to the reliability of the findings, making them applicable for broader clinical considerations.

There are a few limitations in the current study. There may be a possibility of biases in data collection, such as recall bias for transfusion histories and confounders not adjusted for. A small sample size may be another potential limitation. A cross-sectional study design limits the certainty of causality. The appropriate length of follow-up will capture more robust data on complications.

## Conclusions

The prevalence of endocrinopathies in present transfusion-dependent thalassemic cohorts was considerably high, presenting as short stature, impaired glucose tolerance, hypoparathyroidism, and subclinical hypothyroidism. Overt diabetes and pubertal delay were not detected in any of the patients. The study showed a weak positive correlation of endocrinopathies with serum ferritin levels. Hence, irrespective of serum ferritin levels, patients with transfusion-dependent thalassemia can have a considerably high prevalence of endocrine complications. Therefore, close monitoring for endocrine dysfunctions is essential, irrespective of serum ferritin levels, to prevent long-term outcomes. Early interventions, including chelation therapy to manage transfusion-related iron overload, may mitigate complications. Further multicentric studies with larger sample sizes and more robust designs are necessary to validate these findings and inform clinical guidelines.
